# Are rare cancer survivors at elevated risk of subsequent new cancers?

**DOI:** 10.1186/s12885-019-5358-1

**Published:** 2019-02-21

**Authors:** Dianne M. Finkelstein, Nora K. Horick, Ritesh Ramchandani, Kristina L. Boyd, Huma Q. Rana, Brittany L. Bychkovsky

**Affiliations:** 1 Massachusetts General Hospital Biostatistics Center & Department of Biostatistics, Harvard T.H. Chan School of Public Health, 50 Staniford Street, Suite 560, Boston, MA 02114 USA; 2 Massachusetts General Hospital Biostatistics Center, Boston, MA USA; 3 Department of Biostatistics, Harvard T.H. Chan School of Public Health, Boston, USA; 4 Department of Medicine, Dana-Farber Cancer Institute & Harvard Medical School, Boston, MA USA

**Keywords:** Rare cancer, Subsequent cancer risk, Multiple cancers, Survivorship

## Abstract

**Background:**

Although rare cancers account for 27% of cancer diagnoses in the US, there is insufficient research on survivorship issues in these patients. An important issue cancer survivors face is an elevated risk of being diagnosed with new primary cancers. The primary aim of this analysis was to assess whether a history of rare cancer increases the risk of subsequent cancer compared to survivors of common cancers.

**Methods:**

This was a prospective cohort study of 16,630 adults with personal and/or family history of cancer who were recruited from cancer clinics at 14 geographically dispersed US academic centers of the NIH-sponsored Cancer Genetics Network (CGN). Participants’ self-reported cancer histories were collected at registration to the CGN and updated annually during follow-up. At enrollment, 14% of participants reported a prior rare cancer. Elevated risk was assessed via the cause-specific hazard ratio on the time to a subsequent cancer diagnosis.

**Results:**

After a median follow-up of 7.9 years**,** relative to the participants who were unaffected at enrollment, those with a prior rare cancer had a 23% higher risk of subsequent cancer (95% CI: -1 to 52%), while those with a prior common cancer had no excess risk. Patients having two or more prior cancers were at a 53% elevated risk over those with fewer than two (95% CI: 21 to 94%) and if the multiple prior cancers were rare cancers, risk was further elevated by 47% (95% CI: 1 to 114%).

**Conclusion:**

There is evidence suggesting that survivors of rare cancers, especially those with multiple cancer diagnoses, are at an increased risk of a subsequent cancer. There is a need to study this population more closely to better understand cancer pathogenesis.

**Electronic supplementary material:**

The online version of this article (10.1186/s12885-019-5358-1) contains supplementary material, which is available to authorized users.

## Background

Taken as an aggregate, rare cancers account for 27% of the new cancer diagnoses in the United States (US) and 24% of the population of people living with cancer [1]. While there is a growing body of literature about cancer survivors [2], it mainly focuses on each of the more common cancers. Research on the etiology, natural history and treatment of rare cancers is challenged by the small number of patients diagnosed with each cancer and the fact that long-term survival for some rare cancers is low. While five-year survival rates are relatively high for common cancers such as breast (89%) and colorectal (65%), the five-year survival from esophageal, gastric and other rarer gastrointestinal (GI) cancers is less than 30% [3].

An important issue in the care of cancer survivors is prevention of subsequent morbidity, with the development of a new cancer one of the greatest concerns. In fact, cancer survivors now account for 19% of new cancer diagnoses [4]. A study conducted by the Surveillance, Epidemiology, and End Results (SEER) program found that compared to the general population, cancer survivors have a 14% higher risk of developing a new malignancy [2]. Research into the etiology of these subsequent cancers has pointed to the fact that although treatment of the primary cancer is associated with an elevated risk of subsequent cancers [5, 6], the excess new cancer risk is predominantly due to lifestyle and genetic factors [7, 8]. In terms of the absolute excess risk, tobacco- and alcohol-related cancers account for more than 35% of all subsequent malignancies, occurring at sites as diverse as lung, head and neck, and GI tract [2, 8]. Occurrence of subsequent independent primary cancers (i.e. multiple tumors in distinct body systems) suggests an increased cancer susceptibility and sometimes can be attributed to genetic factors. If an individual has an inherited risk, they often tend to develop cancer at a younger age compared to those with sporadic tumors and have a constellation of tumors in sites that suggest a specific genetic syndrome [9]. One confounding factor that further complicates the study of multiple cancers is that often a diagnosis of cancer is followed by intensified screening which can detect second unrelated cancers [10]. Furthermore, as a patient ages their cancer risk increases and this could be a factor in the diagnosis of second cancers, especially for patients whose first cancer was found at a young age. However, only a small minority of people develops second discordant cancers and thus people with multiple cancers may just be a highly susceptible population [9]. It is this population that may lead to better understanding of cancer pathogenesis and the role of genes and environmental factors that influence this susceptibility to cancer. Within this population of susceptible people, patients with rare cancers may have a higher risk of subsequent cancer than those with a common cancer or those with no prior cancer. To date, this risk has not been characterized. To study this, we focus on the risk of subsequent independent primary cancers in individuals who have been diagnosed with a rare cancer compared to those diagnosed with a common cancer and compared to those who are unaffected by cancer. To handle the sparseness of the rare cancers, we use statistical models that allow us to analyze the aggregate of all cancer diagnoses while accounting for the various diagnoses and correcting the variance to reflect the correlation among patients with the same initial cancer type.

## Methods

**Fig. 1:**
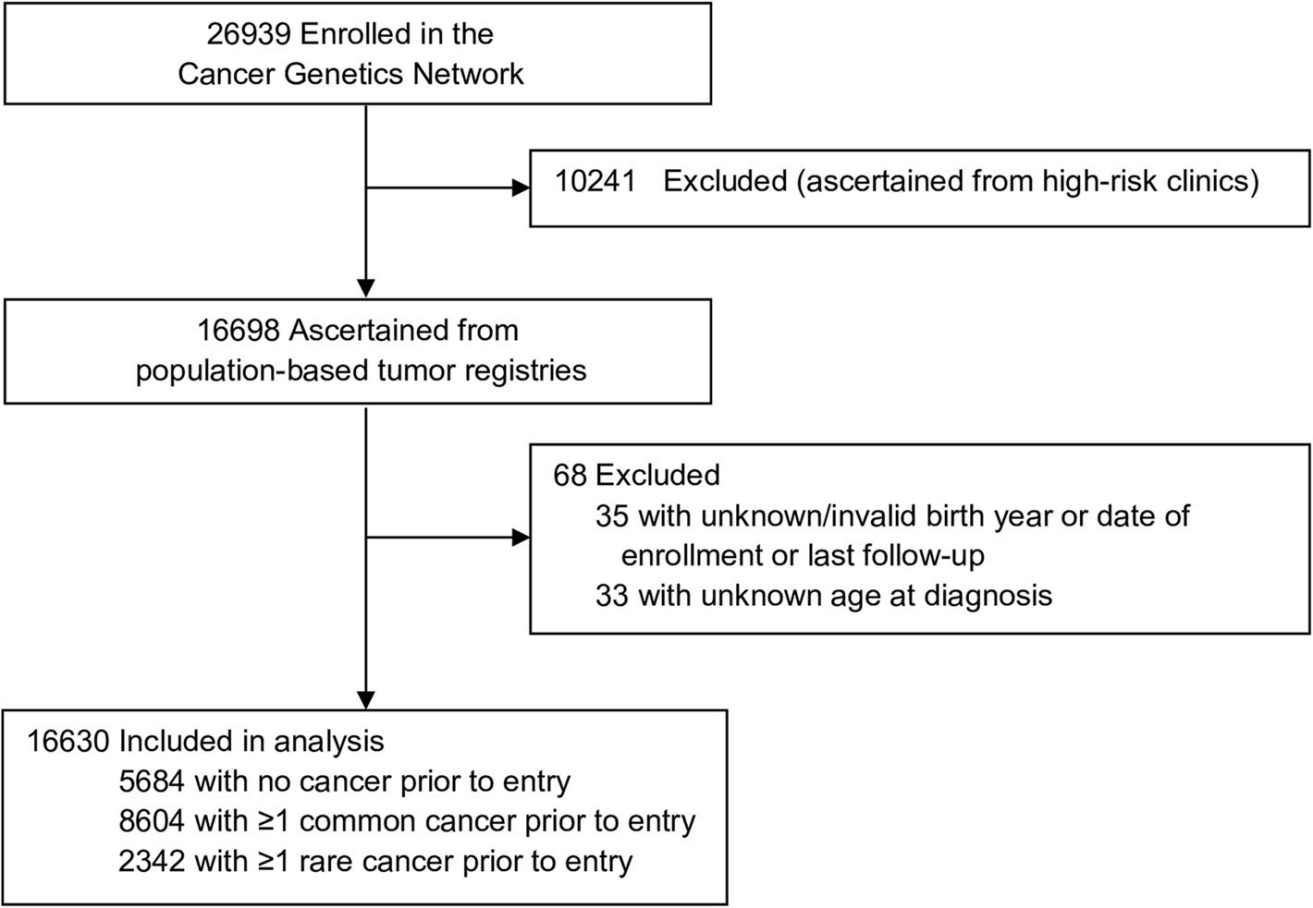
CONSORT diagram

We examined risk of subsequent cancer among adults enrolled in the Cancer Genetics Network (CGN), a national registry of individuals with a personal or family history of cancer established by the National Cancer Institute in 1998 [11]. The CGN enrolled 26,939 adult participants at 14 academic research centers across the US. Institutional Review Boards at each center approved the study, and participants provided informed consent for long-term follow-up; this analysis is restricted to the 16,698 clinic-based recruits (see Fig. 1). At entry, participants provided information on socio-demographic characteristics, personal and four-generation family cancer history, cancer-related medical history, and smoking history. Participants were contacted annually to update baseline information. The median follow-up time is 7.3 years (range: 0–14.9 years). All follow-up ceased in 2012 when the CGN project ended.

**Fig. 2:**
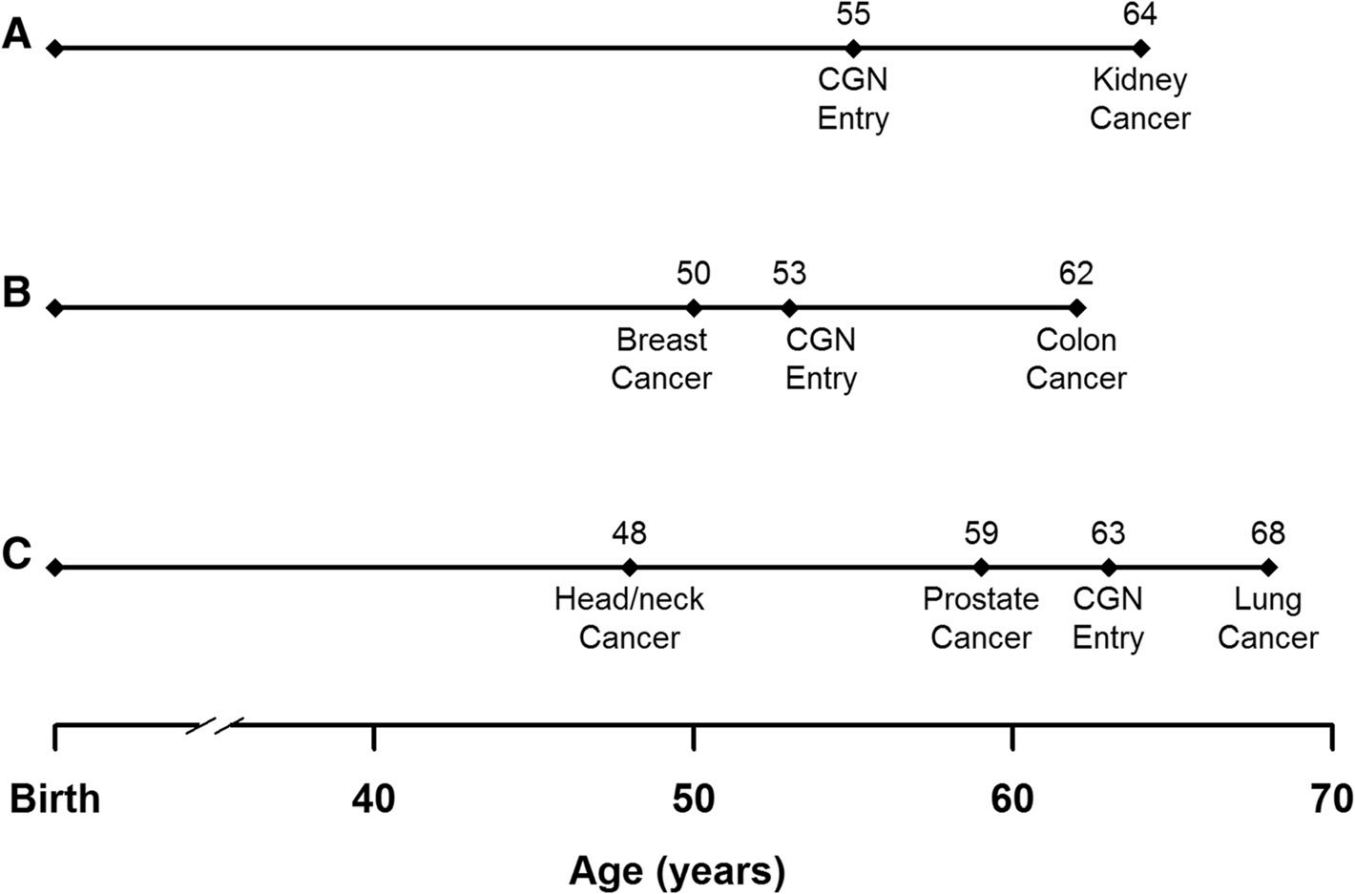
Examples of timing of cancer diagnoses and CGN enrollment for participants with (**a**) no cancer prior to CGN enrollment, (**b**) common cancer (breast) prior to CGN enrollment, and (**c**) rare cancer (head/neck) prior to CGN enrollment. Note that the individual shown in (**c**) also had a common cancer (prostate) prior to CGN enrollment. Participants in the common prior cancer group (**b**) had only common cancers prior to enrollment

The primary outcome of interest was the risk of developing a new primary cancer. Time was measured from CGN enrollment to any type of *new* primary cancer diagnosed during CGN follow-up (event) or last follow-up (censored). Participants were grouped by their cancer history (excluding skin cancer) prior to enrollment (see Fig. 2), including those with no cancer history, those with only common cancers (breast, colorectal, lung, prostate, and melanoma), and those with at least one rare cancer (any non-common cancer). Our definition of rare cancer approximately aligned with the National Institutes of Health (NIH) definition fewer than 15 per 100,000 diagnosed per year in the US [12]. Incidence rates in common cancers are much higher; for example, colorectal and breast cancers have annual incidence rates of 41 and 67 cases per 100,000, respectively. Other factors accounted for in the analysis included age at CGN enrollment, history of tobacco use (current/former versus never), cancer diagnosed in a first-degree relative, prior chemotherapy and radiation (collected in 2011 and available for approximately 70% of participants). Cancer diagnoses that occurred prior to CGN enrollment were confirmed using tumor registries.

Annual hazard rates for subsequent cancer were calculated assuming a Poisson distribution (number of cancers divided by the total years of follow-up). Cox proportional hazards models were used to compare risk of cancer after enrollment, while controlling for other factors. Event times for participants who died during follow-up were treated as censored, resulting in estimates and tests that reflect the “cause-specific hazard” of developing a subsequent cancer in patients who have not yet died. [13] Cox models were fit using a robust sandwich estimator to account for the correlation due to a shared initial cancer [14]. The number of cancers expected to occur during follow-up was calculated using data from the Surveillance, Epidemiology, and End Results (SEER) Program (from 2001) on age/sex-adjusted US incidence of any cancer type multiplied by the length of follow-up for each subject. The expected number of cancers was obtained by adding participants’ expected cancer probabilities, and compared to the observed number of cancers using a chi-square test.

## Results

Participant characteristics are presented in Table 1. Forty-five percent of subjects were over 60 years and only 12% were less than 40 years of age at enrollment. Fifty-nine percent were female and 40% had previously or currently smoked. Participants who were unaffected at enrollment in the CGN were younger and were more likely to report a family history of cancer.

**Table 1:** Demographics, smoking history, and family history by prior cancer group for 16,630 participants in the CGN (1998–2012)

Characteristic	No prior cancers (*N* = 5684)	≥1 prior common cancer (*N* = 8604)	≥1 prior rare cancer (*N* = 2342)	Overall (*N* = 16,630)
Median (range) follow-up time (years)	7.1 (0–14.9)	7.9 (0–14.6)	4.9 (0–12.3)	7.3 (0–14.9)
Age at enrollment (years)				
< 40	1399 (25%)	383 (5%)	269 (12%)	2051 (12%)
40–60	2663 (47%)	3567 (41%)	963 (41%)	7193 (43%)
> 60	1622 (28%)	4654 (54%)	1110 (47%)	7386 (45%)
Female sex	3416 (60%)	4889 (57%)	1584 (68%)	9889 (59%)
Ever used tobacco products	1902 (33%)	3870 (45%)	826 (35%)	6598 (40%)
Any first-degree relative with cancer	5077 (89%)	5849 (68%)	1470 (63%)	12,396 (75%)

The majority of registrants (52%) had only common cancers prior to CGN enrollment, while those remaining had at least one rare cancer prior to enrollment (14%) or no prior cancers (34%). Ten percent (*N* = 867) of the 8604 registrants reporting prior common cancers and 40% (*N* = 945) of the 2342 registrants with a prior rare cancer reported multiple prior cancers. Participants’ prior cancers are summarized in Additional file 1: Table S1. The distribution of cancer types within participants who reported multiple cancers prior to CGN enrollment is given in Additional file 2: Table S2.

We examined the excess risk of subsequent cancer associated with rare cancer in three ways. First, we evaluated the impact of rare cancer on risk for new cancer within our study population. We then compared our study population to the US population to determine whether the effect is sustained. Finally, we analyzed the impact of multiple rare cancers on subsequent cancer risk.

Within our study population, we found that those with a prior rare cancer were at elevated risk relative to the other two groups. This is evident from our initial analysis of the annual (Poisson) cause-specific hazard rates of subsequent cancer. Participants with prior rare cancers had an annual risk of 1.4% compared to 1.3% in those with prior common cancers and 1.0% in those with no prior cancers. We then fit a cause-specific hazard (CSHR) model to allow a direct comparison of the three prior cancer groups (rare, common, and no prior cancer), while adjusting for age, tobacco use and family history. We found that those diagnosed with a prior rare cancer had a 23% higher risk of developing a subsequent cancer during follow-up than those with no prior cancer (CSHR = 1.23, 95% CI: 0.99–1.52, *p* = 0.06), while neither of the other comparisons of the three groups even approached significant differences.

To compare the cancer risk in our study population to the US population (based on SEER data), we compared the observed versus expected cancer rates and these ratios are given by prior cancer history in Table 2. Participants with rare cancers prior to enrollment had more subsequent cancers than expected during follow-up (observed over expected ratio (O/E) = 1.22, *p* = 0.01), while the number of subsequent cancers in the common cancer group was consistent with what would be expected (O/E = 0.99, *p* = 0.82). Participants with no prior cancers reported significantly more cancers than expected (O/E = 1.27, *p* < 0.0001), which is not surprising since these “unaffected” participants were typically recruited due to their family history of cancer, and thus had an elevated cancer risk.

**Table 2:** Relative risk of subsequent compared to US population by prior cancer history

Prior cancer history	*N*	# New cancers	Follow-up years since enrollment	Risk relative to US population^a^
No prior cancer	5684	289	29,680	1.27 (*p* < 0.0001)
≥1 prior common cancer	8604	723	53,618	0.99 (*p* = 0.82)
≥1 prior rare cancer	2342	145	10,352	1.22 (*p* = 0.01)

^a^Relative risk = rate of new cancers observed in study population divided by expected rate in US population based on SEER data

The next analysis focused on the additional risk for subsequent cancer associated with having multiple rare or common cancers before enrollment compared to having one or no prior cancers using a Cox proportional hazards model that adjusted for age, smoking history and prior cancer treatment. Prior cancer history was modeled using six indicator variables —any prior cancer (versus none), two or more prior cancers (versus one or none), any prior rare cancer (versus none), multiple prior rare cancers (versus one or none), any prior common cancer (versus none), and multiple prior common cancers (versus one or none). Only two of the six prior cancer history variables were significant. We found that having two or more prior cancers is associated with a 53% increased risk of subsequent cancer (CSHR = 1.53, 95% CI: 1.21–1.94, *p* = 0.0004). If two or more of the prior cancers is rare, then the participant is at an additional 47% increase in risk for subsequent cancer compared to those with only one or no prior rare cancer (CSHR = 1.47, 95% CI: 1.01–2.14, *p* = 0.04). Thus, having multiple rare cancers is independently predictive of subsequent cancer risk, even after accounting for overall multiplicity of cancers. Neither having two or more prior common cancers (versus one or none) nor having one or more prior common cancers (versus none) was significant in this model. Finally, we did an analysis of the same hypotheses regarding the impact of multiple cancers using a model in which the site of initial cancer was treated as a random effect. The results of this analysis did not change substantially from what is described above.

To assess whether it is reasonable to have grouped cancers in these analyses, we examined the annual cause-specific hazard rates for subsequent cancer within each type of cancer diagnosed before enrollment as well as the cause-specific hazard ratios comparing subsequent cancer risk for each cancer to that for prior common and no prior cancer (see Additional file 3: Table S3). There is little evidence of differences between the various rare and common cancer types, and this stratified analysis reflects a similar result to what was reported in the analysis that combined all rare and all common cancer types.

## Discussion

Cancer survivors have an increased risk of developing a subsequent cancer compared to the general population and individuals with multiple primaries now account for a substantial fraction of the newly diagnosed cancers [15]. While these results have been established in more common cancers, the risk of a second primary for patients with rare cancers has not been addressed until now. We found that participants with at least one rare cancer had a 23% increased risk of a subsequent cancer diagnosis compared with individuals with no prior cancer diagnosis, and those with at least two rare cancers had a 47% increased risk of a subsequent cancer diagnosis compared to those with fewer than two prior rare cancers.

This study had several potential weaknesses. First, since we used data from the CGN, there was likely a selection bias in our cohort since people who participated may have been individuals with heightened cancer awareness and family history which could have resulted in inaccurate risk estimates. For example, those without prior cancer were probably at greater risk of cancer than the comparison general population. Second, this is self-reported information so participants reporting multiple cancers may not in fact have a new cancer diagnosis but were reporting a recurrence or metastasis of a previously diagnosed primary cancer. We tried to avoid this issue in the study by asking for participants to only report independent primaries or new cancer diagnoses. Third, since this was a long-term study, we do not know how much our results were impacted by attrition (including death)– our findings may be subject to under-reporting bias. For example, patients with a history of pancreatic cancer would not likely be in the CGN as they would have died soon after the cancer was diagnosed. Fourth, we did not stratify our study by whether a participant had known cancer susceptibility mutation as this information was not available so some of our results may be a reflection of known and reported syndromes such as BRCA which is associated with excess breast and ovarian cancer risk. Finally, it has been noted that treatment for prior cancer could be associated with an elevated risk of subsequent cancer [7, 8]. However, our data on prior treatment were self-reported and incomplete, and we were not able to thoroughly assess the impact of treatment on our results. Despite these limitations in our study, the findings have value since they prompt many questions about cancer pathogenesis that could be explored in this high-risk cohort.

Historically, patients with rare cancers have not been the focus of cancer research since it is difficult to enroll a sufficient number of patients. It is in this context that our findings are significant as they highlight the need for more research on rare cancers. The statistical methods we apply are promising as they allow us to aggregate patients over multiple cancer sites but still take into account the underlying differences in hazard associated with these different cancers and permit us to include very rare cancers. There is always a trade-off required between aggregating to increase power and analyzing within homogeneous groups to clarify interpretation, but for the study of rare cancers, it is often infeasible to consider each disease alone.

Traditionally it was thought that only 5–10% of all cancer diagnoses are explained by an inherited cancer syndrome [16]. However, a recent study found that 17.5% of individuals with advanced cancers were found to have an underlying cancer genetic predisposition [17]. We are beginning a transition in oncology focusing less on monogenic inherited cancer syndromes and instead emphasizing research that is exploring how polygenic disease determinants including single-nucleotide polymorphisms (SNPs), the immune system, epigenetics, the proteome and the microbiome may influence an individual’s cancer risk [18–20]. We believe that in this setting, there is a need to explore the pathogenesis of rare cancers.

## Conclusions

We have found that patients with a history of a rare cancer are at elevated risk for a future independent cancer. The clinical significance of this is that rare cancer survivors could benefit from more intensive measures of prevention and surveillance. The scientific significance of this work is that future research needs to focus specifically on patients with two or more rare cancer diagnoses. This should include the rapidly emerging information available from mutational panels and newly discovered biomarkers. Also, as new tumor classifications are available, the analyses in this paper should be repeated, as cancer care is an evolving field. With a better understanding of rare cancer pathogenesis and the natural course of these diseases, we will better be able to risk stratify cancer survivors in a way that we can personalize prevention and screening strategies.

## Additional files

Additional file 1: **Table S1.** Participants’ prior cancers for those with rare versus common cancers. Tabulation of participants’ prior cancers grouped by whether the participants had prior rare cancers versus only prior common cancers. (DOCX 13 kb)

Additional file 2: **Table S2.** Distribution of prior cancer types among those with multiple prior cancers. Tabulation of prior cancer types among the 1812 participants who reported multiple cancers prior to CGN enrollment. (DOCX 12 kb)

Additional file 3: **Table S3.** Hazard rates and ratios for subsequent cancer by type of prior cancer. Annual Poisson cause-specific hazard rates and cause-specific hazard ratios for subsequent cancer risk within subgroups defined by participants’ cancer type prior to CGN enrollment. (DOCX 14 kb)

## Abbreviations

BRCA: Breast cancer gene
CGN: Cancer Genetics Network
CSHR: Cause-specific hazard ratio
N: Number
NIH: National Institutes of Health
O/E: Number observed divided by number expected
SEER: Surveillance, Epidemiology, and End Results Program
SNP: Single nucleotide polymorphism
US: United States

## References

[1] Gatta G, van der Zwan JM, Casali PG, Siesling S, Dei Tos AP, Kunkler I (2011). Rare cancers are not so rare: the rare cancer burden in Europe. Eur J Cancer.

[2] Curtis RE. New malignancies among cancer survivors : SEER cancer registries, 1973–2000. Bethesda, Md.: U.S. Dept. of Health and Human Services, National Institutes of Health, National Cancer Institute; 2006. viii, 492 p. p.

[3] Wilbur J (2015). Surveillance of the adult cancer survivor. Am Fam Physician.

[4] Morton LM, Onel K, Curtis RE, Hungate EA, Armstrong GT. The rising incidence of second cancers: patterns of occurrence and identification of risk factors for children and adults. Am Soc Clin Oncol Educ Book. 2014:e57–67. 10.14694/EdBook_AM.2014.34.e57 24857148

[5] Ng J, Shuryak I (2015). Minimizing second cancer risk following radiotherapy: current perspectives. Cancer Manag Res.

[6] Network NCC. Understanding Your Risk of Developing Secondary Cancers [Available from: https://www.nccn.org/patients/resources/life_after_cancer/understanding.aspx.

[7] Berrington de Gonzalez A, Curtis RE, Kry SF, Gilbert E, Lamart S, Berg CD (2011). Proportion of second cancers attributable to radiotherapy treatment in adults: a cohort study in the US SEER cancer registries. Lancet Oncol.

[8] Wood ME, Vogel V, Ng A, Foxhall L, Goodwin P, Travis LB (2012). Second malignant neoplasms: assessment and strategies for risk reduction. J Clin Oncol.

[9] Garber JE, Offit K (2005). Hereditary cancer predisposition syndromes. J Clin Oncol.

[10] Choi M, Craft B, Geraci SA (2011). Surveillance and monitoring of adult cancer survivors. Am J Med.

[11] Anton-Culver H, Ziogas A, Bowen D, Finkelstein D, Griffin C, Hanson J (2003). The Cancer genetics network: recruitment results and pilot studies. Community Genet.

[12] Department of Health and Human Services (US). Office of Rare Diseases NIoH. Annual report on the rare diseases research activities at the National Institutes of Health. 2005/2006.

[13] Varadhan R, Weiss CO, Segal JB, Wu AW, Scharfstein D, Boyd C (2010). Evaluating health outcomes in the presence of competing risks: a review of statistical methods and clinical applications. Med Care.

[14] Lee EW, Wei LJ, Amato DA, Leurgans S, Klein JP, Goel PK (1992). Cox-type regression analysis for large numbers of small groups of correlated failure time observations. Survival analysis: state of the art.

[15] Amer MH (2014). Multiple neoplasms, single primaries, and patient survival. Cancer Manag Res.

[16] Nagy R, Sweet K, Eng C (2004). Highly penetrant hereditary cancer syndromes. Oncogene.

[17] Mandelker D, Zhang L, Kemel Y, Stadler ZK, Joseph V, Zehir A (2017). Mutation detection in patients with advanced Cancer by universal sequencing of Cancer-related genes in tumor and Normal DNA vs guideline-based germline testing. JAMA.

[18] Institute NC. Understanding SNPs and Cancer [Available from: http://www.auburn.edu/academic/classes/biol/3020/iActivities/CGAP2/SNPS.pdf.

[19] Blancafort P, Jin J, Frye S (2013). Writing and rewriting the epigenetic code of cancer cells: from engineered proteins to small molecules. Mol Pharmacol.

[20] Corthay A (2014). Does the immune system naturally protect against cancer?. Front Immunol.

