# Supplementary material for: Are rare cancer survivors at elevated risk of subsequent new cancers?
Source: BMC Cancer. 2019 Feb 21;19:166. doi: 10.1186/s12885-019-5358-1 (PMC6385466; doi:10.1186/s12885-019-5358-1)
Supplement: Supplementary file 1 — Table S1. Participants’ prior cancers for those with rare versus common cancers. Tabulation of participants’ prior cancers grouped by whether the participants had prior rare cancers versus only prior common cancers. (DOCX 13 kb) [file 12885_2019_5358_MOESM1_ESM.docx]

**Additional file 1: Table S1. Participants’ prior cancers for those with rare versus common cancers^1^**

|  | **Pts with ≥1 prior common cancer, # (%)**  **(n=8604)** | **Pts with ≥1 prior rare cancer, # (%)**  **(n=2342)** |
| --- | --- | --- |
| **Common** |  |  |
| Breast | 3956 (46) | 400 (17) |
| Prostate | 2744 (32) | 190 (8) |
| Colon/rectum | 1191 (14) | 153 (7) |
| Melanoma | 967 (11) | 77 (3) |
| Lung | 187 (2) | 51 (2) |
| **Rare** |  |  |
| Gynecologic |  | 746 (32) |
| Ovary & fallopian tube & peritoneum | - | 428 (18) |
| Uterus/endometrium | - | 193 (8) |
| Cervix | - | 133 (6) |
| Other female genital | - | 17 (1) |
| Gastrointestinal | - | 481 (21) |
| Pancreas | - | 203 (9) |
| Kidney & renal pelvis & ureter | - | 156 (7) |
| Liver | - | 60 (3) |
| Stomach | - | 30 (1) |
| Esophagus | - | 11 (<1) |
| Small intestine | - | 10 (<1) |
| Gallbladder & biliary tract | - | 9 (<1) |
| Appendix | - | 6 (<1) |
| Anus | - | 4 (<1) |
| Thyroid & other endocrine glands | - | 484 (21) |
| Thyroid | - | 480 (21) |
| Adrenal & other endocrine glands | - | 5 (<1) |
| Hematologic | - | 296 (13) |
| Genitourinary | - | 208 (9) |
| Bladder & other urinary | - | 159 (7) |
| Testis | - | 48 (2) |
| Other male genital | - | 1 (<1) |
| Sarcoma | - | 63 (3) |
| Head/neck | - | 51 (2) |
| Neurologic | - | 41 (2) |
| Other | - | 2 (<1) |
| Unknown |  | 86 (4) |
| ^1^ Participants with multiple prior cancers are included in multiple rows corresponding to each type of prior cancer; as a result, number/percent across rows sum to greater than column totals/100%. | | |
