# Supplementary material for: Are rare cancer survivors at elevated risk of subsequent new cancers?
Source: BMC Cancer. 2019 Feb 21;19:166. doi: 10.1186/s12885-019-5358-1 (PMC6385466; doi:10.1186/s12885-019-5358-1)
Supplement: Supplementary file 2 — Table S2. Distribution of prior cancer types among those with multiple prior cancers. Tabulation of prior cancer types among the 1812 participants who reported multiple cancers prior to CGN enrollment. (DOCX 12 kb) [file 12885_2019_5358_MOESM2_ESM.docx]

**Additional file 2: Table S2. Distribution of prior cancer types
among those with multiple prior cancers.**

| **Prior cancer type** | **# (%)** |
| --- | --- |
| adrenal & other endocrine glands | 3 (<1) |
| anus | 3 (<1) |
| appendix | 5 (<1) |
| bladder & other urinary | 125 (3) |
| breast | 1389 (35) |
| cervix | 85 (2) |
| colorectal | 499 (13) |
| esophagus | 5 (<1) |
| female genital | 12 (<1) |
| gallbladder & biliary tract | 5 (<1) |
| head/neck | 38 (1) |
| hematologic | 184 (5) |
| kidney & renal pelvis & ureter | 80 (2) |
| liver | 29 (1) |
| lung | 123 (3) |
| male genital | 1 (<1) |
| melanoma | 348 (9) |
| neurologic | 21 (1) |
| other | 2 (<1) |
| ovary & fallopian tube & peritoneum | 148 (4) |
| pancreas | 30 (1) |
| prostate | 415 (11) |
| sarcoma | 42 (1) |
| small intestine | 9 (<1) |
| stomach | 16 (<1) |
| testis | 17 (<1) |
| thyroid | 123 (3) |
| unknown | 48 (1) |
| uterus/endometrium | 130 (3) |
| **Total** | 3935 (100) |
