# Supplementary material for: Are rare cancer survivors at elevated risk of subsequent new cancers?
Source: BMC Cancer. 2019 Feb 21;19:166. doi: 10.1186/s12885-019-5358-1 (PMC6385466; doi:10.1186/s12885-019-5358-1)
Supplement: Supplementary file 3 — Table S3. Hazard rates and ratios for subsequent cancer by type of prior cancer. Annual Poisson cause-specific hazard rates and cause-specific hazard ratios for subsequent cancer risk within subgroups defined by participants’ cancer type prior to CGN enrollment. (DOCX 14 kb) [file 12885_2019_5358_MOESM3_ESM.docx]

**Additional file 3: Table S3. Hazard rates and ratios for subsequent cancer by type of prior cancer^1^**

| **Prior cancer type^2^** | **N** | **# Events** | **Annual Poisson cause-specific hazard rate^3^** | **Cause-specific hazard ratio^4^,  rare vs common** | **Cause-specific hazard ratio^4^,  row vs unaffected** |
| --- | --- | --- | --- | --- | --- |
| liver | 60 | 6 | 3.1% | 2.24* | 2.63* |
| sarcoma | 64 | 7 | 2.6% | 1.87 | 2.19* |
| lung | 250 | 18 | 2.3% | - | 1.70 |
| bladder & other urinary | 172 | 21 | 2.3% | 1.45 | 1.70* |
| stomach | 31 | 3 | 2.2% | 1.59 | 1.86 |
| hematologic | 315 | 30 | 2.0% | 1.47* | 1.72** |
| neurologic | 41 | 3 | 2.0% | 1.64 | 1.91 |
| melanoma | 1097 | 87 | 1.8% | - | 1.36 |
| colorectal | 1416 | 130 | 1.7% | - | 1.86** |
| ovary & fallopian tube & peritoneum | 439 | 33 | 1.6% | 1.29 | 1.50* |
| breast | 4799 | 479 | 1.5% | - | 1.29 |
| kidney & renal pelvis & ureter | 160 | 11 | 1.5% | 0.99 | 1.15 |
| uterus/endometrium | 195 | 13 | 1.2% | 0.82 | 0.96 |
| prostate | 2949 | 231 | 1.2% | - | 1.42 |
| testis | 49 | 3 | 1.2% | 1.10 | 1.28 |
| thyroid | 486 | 18 | 0.9% | 0.83 | 0.97 |
| head/neck | 54 | 2 | 0.8% | 0.51 | 0.59 |
| cervix | 136 | 6 | 0.8% | 0.60 | 0.70 |
| pancreas | 203 | 1 | 0.4% | 0.34 | 0.40 |
| ^1^ Results are presented in descending order by hazard rate. Only cancers with n ≥ 30 are presented.  ^2^ Participants with multiple prior cancers are included for each prior cancer type.  ^3^ Annual Poisson cause-specific hazard rates are estimates of the percentage of participants with a given prior cancer type who will develop a new cancer per year  ^4^ Hazard ratios are obtained from Cox models and adjusted for age at enrollment; * = p < 0.05, ** = p < 0.01 | | | | | |
